# Clinical outcomes of various types of revision surgeries after unicompartmental knee arthroplasty failure

**DOI:** 10.1186/s12891-023-06413-x

**Published:** 2023-04-18

**Authors:** Shih-Hui Peng, Chun-Chieh Chen, Sheng-Hsun Lee, Yu-Chih Lin, Jui-Fan Chiang, Szu-Yuan Chen, Chih-Chien Hu, Yuhan Chang, Pang-Hsin Hsieh, Hsin-Nung Shih, Chih-Hsiang Chang

**Affiliations:** 1 Department of Orthopedic Surgery, Chang Gung Memorial Hospital, Linkou, Taiwan, No. 5, Fuxing St., Guishan Dist., Taoyuan City, Taiwan; 2 College of Medicine, Chang Gung University, Taoyuan, Taiwan; 3 Bone and Joint Research Center, Chang Gung Memorial Hospital, Linkou, Taiwan; 4 Graduate Institute of Clinical Medical Sciences, College of Medicine, Chang Gung University, Taoyuan, Taiwan

**Keywords:** Failed unicompartmental knee arthroplasty, Aseptic loosening, Revision surgery

## Abstract

**Purpose:**

The advantages of unicompartmental knee arthroplasty (UKA) have led to the procedure being increasingly performed worldwide. However, revision surgery is required after UKA failure. According to the literature review, the choice of implant in revision surgery remains a debatable concern. This study analyzed the clinical results of different types of prostheses used in treating failed UKA.

**Materials and methods:**

This is a retrospective review of 33 failed medial UKAs between 2006 and 2017. Demographic data, failure reason, types of revision prostheses, and the severity of bone defects were analyzed. The patients were classified into three groups: primary prosthesis, primary prosthesis with a tibial stem, and revision prosthesis. The implant survival rate and medical cost of the procedures were compared.

**Results:**

A total of 17 primary prostheses, 7 primary prostheses with tibial stems, and 9 revision prostheses were used. After a mean follow-up of 30.8 months, the survival outcomes of the three groups were 88.2%, 100%, and 88.9%, respectively (P = 0.640). The common bone defect in tibia site is Anderson Orthopedic Research Institute [AORI] grade 1 and 2a (16 versus 17). In patients with tibial bone defects AORI grade 2a, the failure rates of primary prostheses and primary prostheses with tibial stems were 25% and 0%, respectively.

**Conclusions:**

The most common cause for UKA failure was aseptic loosening. The adoption of a standardized surgical technique makes it easier to perform revision surgeries. Primary prostheses with tibial stems provided higher stability, leading to a lower failure rate due to less risk of aseptic loosening in patients with tibial AORI grade 2a. In our experience, we advise surgeons may try using primary prostheses in patients with tibial AORI grade 1 and primary prostheses with tibial stems in patients with tibial AORI grade 2a.

## Introduction

Unicompartmental knee arthroplasty (UKA) is used to address pain and functional problems associated with osteoarthritis (OA) and osteonecrosis (ON) of the knee and is a more favorable option for treating unicompartmental OA or ON than high tibial osteotomy due to faster recovery, superior pain relief, and ease of revision [1–4]. The advantages of UKA over total knee arthroplasty (TKA) are well known and include the preservation of bone and soft tissue, retention of the anterior cruciate ligament, earlier and easier recovery, superior functional outcome, and less requirement for blood transfusion in the immediate postoperative period [5–8].

In 2013, Foran et al. reported the long-term outcomes and failure modes of UKA, with a survival rate of 93% at 15 years and 90% at 20 years after UKA [9]. Only 4 of 62 patients (9.7%) were revised to TKA, and all of them were revised because of reasons other than septic or aseptic loosening. Improved implants, careful patient selections, and developments in surgical techniques have made UKA outcomes comparable to those of TKA. However, a steady increase in UKAs has led to an increase in the number of revision procedures [7, 10, 11].

The causes of UKA failure include aseptic loosening, infection, polyethylene wear, periprosthetic fracture, and advanced OA [12–14]. With the adoption of a standardized technique, revision surgery for failed UKA can be achieved with TKA despite the loss of bone stock and anatomical landmarks [15]. According to literature review, various types of revision surgeries are performed for failed UKAs by using primary prostheses, primary prostheses with tibial stems, and revision prostheses [16, 17]. However, little information has been published on the addressing the survival outcome of these procedures. And we assumed that the infection rate after surgery with revision prostheses may be high because of the requirement for more surgical invasion. Compared with revision prostheses and primary prostheses with tibial stems, primary prostheses offer a superior range of motion for patients; however, their durability remains debatable.

This retrospective study reported the clinical outcomes of TKA after UKA failure at our institution and compared the pros and cons of primary prostheses, primary prostheses with tibial stems, and revision prostheses. We hypothesized that revision of UKA to TKA is possible and that primary prostheses with tibial stems exhibit a lower infection rate than revision prostheses and are more stable than primary prostheses.

## Materials and methods

### Study design

This study was approved by the Biomedical Institutional Review Board of our hospital (103-3539B). Patients with failed UKA were identified using the healthcare information system (HIS) of our hospital between 2006 and 2017. This retrospective study reviewed the reasons for revision on the basis of patients’ symptoms, documented history, diagnostic images, and intraoperative findings. Patients undergoing any revision surgery after UKA failure were included. Patients with incomplete baseline data and who were lost to follow-up were excluded. During the study period, 46 consecutive patients were converted to TKA. Of them, 13 patients were excluded due to incomplete data, and 33 were included. Of them, 17 received primary prostheses, 7 received primary prostheses with tibial stems, and 9 received revision prostheses. The mean follow-up time was 30.8 months.

### Surgical method

All revision surgeries were performed by arthroplasty surgeons with experience in more than 100 cases including TKA and UKA per year at our referral arthroplasty center. The patients were followed up from the time of revision by using the HIS. The revision surgeries were performed by following a standard technique. First, the femoral component and polyethylene insert were removed. Subsequently, the thickness of the distal femoral cut was measured on the basis of the lateral femoral condyle. Second, the tibial component was removed, and a tibial cut was made by measuring lateral tibial plateau thickness. Finally, femoral posterior resection was performed relative to the epicondylar line, and the medial and lateral compartment flexion gap was balanced. Bone defects, soft tissue condition, and stability were assessed to select primary prostheses, primary prostheses with tibial stems, or revision prostheses. The prostheses that we used in revision surgeries included Zimmer LPS, Zimmer LCCK, United U2, United PSA, Stryker NRG, Osteonics Scorpio and Depuy RP based on surgeons’ selection.

### Outcome measurement

Patient demographics, including sex, age, preoperation (pre-OP) and postoperation (post-OP) Knee Society Scores (KSSs), and femoral and tibial Anderson Orthopedic Research Institute (AORI) classification, were recorded at the time of UKA and revision surgery. Moreover, whether the patients received bone grafts was recorded. The dates of primary implantation and revision surgery were obtained. All causes of early and late failure were documented (Table 1). Other compartment arthritis was defined as arthritis in the lateral and/or patellofemoral compartment. In addition, a detailed radiographic analysis after revision surgery was performed to check the prosthesis condition and alignment. The survival rate of primary prostheses was compared with that of revision prostheses to evaluate their durability.

**Table 1:** Baseline characteristics of patients and procedures

	Primary prostheses (n = 17)	Primary prostheses with tibia stems (n = 7)	Revision prostheses (n = 9)	*P value*
Age (years)	67.94 ± 9.42	72.00 ± 8.17	64.44 ± 10.26	0.779
Female	14 (82.4%)	6 (85.7%)	9 (88.9%)	0.905
F/U time (months)	27.35 ± 27.41	24.00 ± 13.89	42.78 ± 25.82	0.156
Duration of Procedure (min)	128.35 ± 30.51	152.86 ± 26.45	139.67 ± 36.09	0.803
Pre–OP KSS score	67.24 ± 5.90	62.29 ± 14.67	65.67 ± 4.36	0.052
Tibial AORI = 1	9 (52.9%)	2 (28.6%)	5 (55.6%)	0.490
Tibial AORI = 2a	8 (47.1%)	5 (71.4%)	4 (44.4%)	0.490
Femoral AORI = 1	17 (100%)	7 (100%)	9 (100%)	1.000

### Statistical analysis

Descriptive statistics are presented as numbers (of occurrences), percentages or means, standard deviations, and ranges. A chi-squared test and one-way analysis of variance were used to calculate the differences between the groups. Statistical analyses were performed using SPSS 22.0 (SPSS Inc., Chicago, IL, USA). The threshold for statistical significance was set at P = 0.05.

## Results

Demographic data, including age, sex, follow-up period, procedure duration, pre-OP KSS, and bone defect, were documented. No significant difference in demographic data was observed among the three groups (Table 2). Bone defects in tibia site were AORI grade 1 and 2a in medial site (16 versus 17), and the defects in femoral site were all AORI grade 1. The most common reason for the revision was aseptic loosening (18 of 33), followed by periprosthetic joint infection and insert wear. Only two cases received revision surgery due to advanced OA of other compartments (Table 1).

**Table 2:** Causes of UKA failure

Causes	Primary prostheses	Primary prostheses with tibial stems	Revision prostheses	Total
Aseptic loosening	12	3	3	18
Infection		2	3	5
Wearing	4		1	5
Periprosthetic fracture		2	1	3
Advanced OA	1		1	2

### Survival outcomes between primary prostheses, primary prostheses with tibial stems, and revision prostheses

A total of 24 patients received primary prostheses after UKA failure. Of these, two procedures failed again due to aseptic loosening of the tibial component, leading to the requirement of revision prostheses. Among 9 patients who received revision prostheses after a failed UKA, failure due to periprosthetic joint infection was observed in 1 patient.

**Fig. 1:**
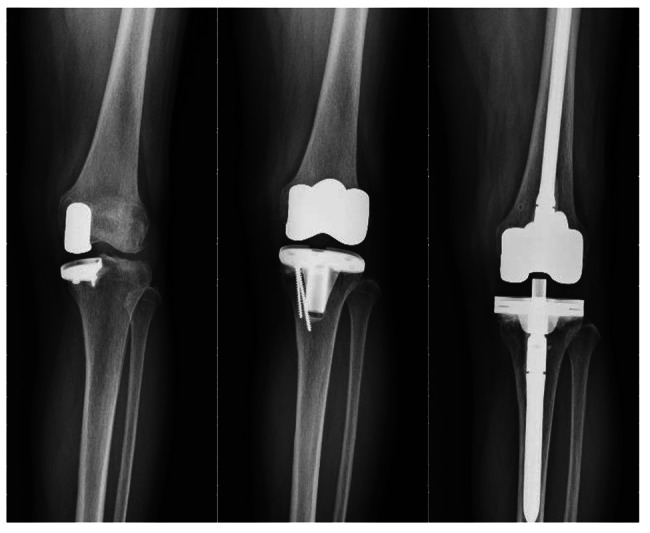
Anteroposterior radiograph of Patient A’s knee. Unicompartmental knee arthropasty (UKA) was performed in the past (left), procedure with primary prostheses and bone graft after UKA failure (center), and procedure with revision prostheses after primary prostheses failure due to tibia site loosening (right)

**Fig. 2:**
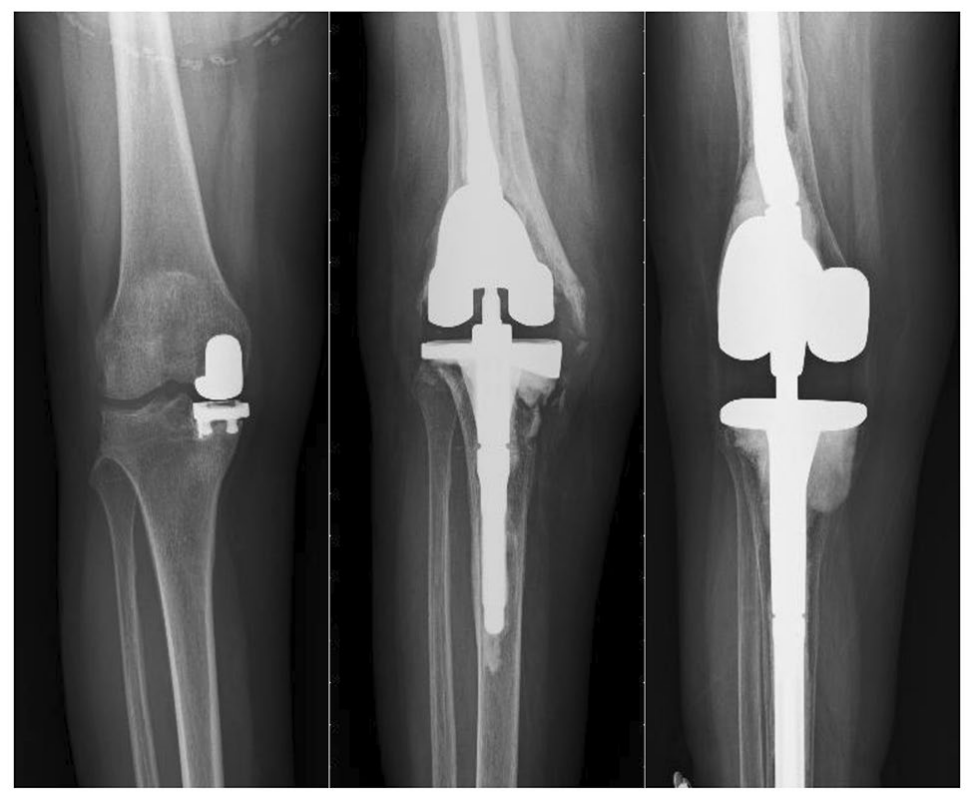
Anteroposterior radiograph of Patient B’s knee. Unicompartmental knee arthropasty (UKA) was performed in the past (left), procedure with revision prostheses because of bone defect after UKA failure (center), and procedure with revision prostheses after the failure of the original prosthesis because of periprosthetic joint infection (right)

Patient A was a 60-year-old woman with a body mass index (BMI) of 26.60 kg/m^2^, who received revision prostheses on her left knee in December 2011; the procedure failed in February 2016 because of tibia site prosthesis loosening. The pre-OP and post-OP KSSs were 65 and 75, respectively. The femoral and tibial AORI grades were 1 and 2a, respectively, and the bone defect was treated using a bone graft (Fig. 1). Patient B was a 60-year-old woman with a BMI of 32.68 kg/m^2^, who received revision prostheses on the right knee in February 2010; periprosthetic joint infection occurred in May 2012. The pre-OP and post-OP KSSs were 60 and 80, respectively. The femoral and tibial AORI grades were 1 and 2a, respectively, with a tibial metal augment used in revision prostheses (Fig. 2).

We further compared the survival outcomes between the groups. The chi-squared test (Table 3) revealed no significant difference in survival outcome between the primary prosthesis, primary prosthesis with a tibial stem, and revision prosthesis groups. However, when comparing primary prostheses and primary prostheses with tibial stems (with a tibial AORI grade of 2a), primary prostheses with tibial stems provided higher stability. Although P values did not reach statistical significance, the success rate was 75% for primary prostheses and 100% for primary prostheses with tibial stems (Table 4). No failure was observed in each group for a tibial AORI grade of 1.

**Table 3:** Survival outcomes between primary prostheses, primary prostheses with tibial stems, and revision prostheses

	Primary prostheses (n = 17)	Primary prostheses with tibial stems (n = 7)	Revision prostheses (n = 9)	*P value*
Success	15 (88.2%)	7 (100.0%)	8 (88.9%)	0.640
Failure	2 (11.8%)^1^	0 (0.0%)	1 (11.1%)^2^	

^1^ The cause of failure of primary prostheses was aseptic loosening.

^2^ The cause of failure of revision prostheses was infection.

**Table 4:** Failure rates of primary prostheses and primary prostheses with tibial extended rods in patients with tibial AORI grade 2a

	Primary prostheses (n = 8)	Primary prostheses with tibial stems (n = 5)	Revision prostheses (n = 4)	*P* *value*
Success	6 (75.0%)	5 (100.0%)	3 (75.0%)	0.520
Failure	2 (25.0%)	0 (0.0%)	1 (25.0%)	

### Procedure duration

The procedure duration was the interval between the Procedure/Surgery Start Time and the Procedure/Surgery Finish Time, as defined by the Association of Anesthesia Clinical Directors.

**Fig. 3:**
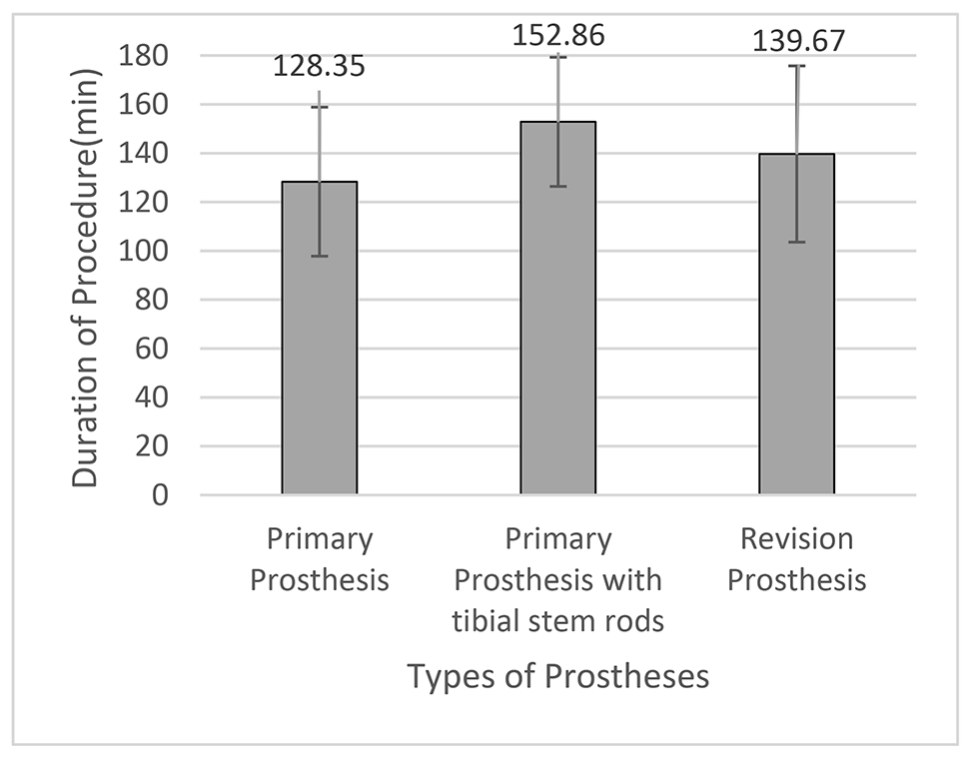
Procedure duration of primary and revision prostheses. The mean procedure duration was longest in the primary prosthesis with a tibial stem group. But no significant difference in procedure duration was observed between three groups

The alignment of primary prostheses without the use of stem rods and the fixation of stem rods with the use of revision prostheses are time-consuming. Therefore, we analyzed the procedure duration of UKAs performed using primary prostheses, primary prostheses with tibial stems, and revision prostheses in our hospital. The mean procedure duration was 128.35 min in the primary prosthesis group, 152.86 min in the primary prosthesis with a tibial stem group, and 139.67 min in the revision prosthesis group. No significant difference in procedure duration was observed between the groups, with a P value of above 0.05 (Fig. 3).

## Discussion

UKA prostheses are being developed for a long time; however, stricter indications may lower the revision rates. In the past 20 years, the number of UKAs has increased, leading to an increased number of revisions. A study reported that the revision rate of UKA ranges from 3.4 to 13%, with the most frequent causes of UKA failure being OA progression and aseptic loosening [15]. Some studies have described the technique and provided surgical tips for revisions after UKA failure [16, 17]. However, little information has been published on the survival outcome of procedures with primary prostheses and revision prostheses after UKA failure. Our study described the surgical tips for revision surgery, the reasons for UKA failure, and the survival rates of procedures with primary prostheses, primary prostheses with tibial stems, and revision prostheses. In our study, the most frequent cause of UKA failure was aseptic loosening rather than OA progression, which is contradictory to the results of a previous study [15]. This may be because of the stricter indication adopted in this study for UKA. If radiographic or intraoperative findings suggest other compartment OA, the patient is converted to TKA immediately. In addition, insert wear and periprosthetic joint infection caused UKA failure, which is consistent with the results of previous studies [7, 18, 19].

### Management of bone defects

Châtain F. included 54 French patients and analyzed technical difficulties in revision TKAs in patients with unicompartmental femorotibial prostheses and concluded that tibial bone loss is more frequent; however, the correction of femoral bone loss is more challenging [20]. Moreover, in our study, femoral AORI grades were all 1; therefore, the correction of femoral bone loss was not required. In such cases, treatment should focus on the management of tibial bone loss. The tibial side bone loss may be caused by the native tibial bone cut. If the defect is greater than the line orthogonal to the mechanical axis drawn 10 mm below the joint of the unaffected compartment, a wedge, bone graft, or tibial stem may be required [19]. Different methods for managing tibial bone loss include bone grafts with screw fixation, procedures with primary prostheses with tibial stems, and procedures using revision prostheses with augments. For tibial bone loss with AORI grade 1, the survival rate is satisfactory when using primary prostheses with some morselized bone graft harvest from the native joint compartment.

We further analyzed patients with tibial bone loss classified as AORI grade 2a. The results indicated that the survival rate was the highest in the primary prosthesis with a tibial stem group. Only 2 of 8 patients with primary prostheses developed tibial site early aseptic loosening in the follow-up period, whereas 1 of 4 patients with revision prostheses developed a periprosthetic joint infection and required two-stage exchange arthroplasty. In our study, revision prostheses were used in 27% of cases, which is consistent with the results of a previous study [21]. Leta et al. reported that the overall rate of rerevision from UKA to TKA was 12%, which is close to the 11% value obtained in this study [22]. However, we discovered that the rerevision rate was higher (17%) in the AORI grade 2a group. The main reason for re-revision was tibial loosening, and the only patient who developed a periprosthetic joint infection in our study used a revision prosthesis. Leta et al. also discovered that deep infection is higher when stems and a more constrained prosthesis are used. Two studies on the use of screws and cement in primary or revision TKA were published by Berend ME et al. in 2014 and 2015 [23, 24]. They concluded that the performance of the knees with tibial defects and screws was similar to that of those without defects; moreover, the procedure involved substantially lower cost than the alternatives. In our study, 17 patients had bone defects with tibial AORI grade 2a, and 16 of them received bone grafts or metal augments. Radiographic findings did not reveal early aseptic loosening or bone graft absorption. We believe that compared with revision prostheses, primary prostheses with bone grafts and tibial stems offer a smaller degree of surgical invasion, share the load and protect bone grafts, and reduce the risk of infection.

### Medical cost

A study investigated the medical costs involved in primary and revision TKA and suggested that efforts should be made to reduce the high costs of revision prostheses [23]. Increased costs associated with demographic factors and comorbidities may put providers at financial risk and may jeopardize health-care access for patients in greatest need [25]. The study reported that the largest proportion of costs in both primary and revision prostheses was for room and boarding (28% vs. 23%), followed by operating room (22% vs. 17%), and prostheses (13% vs. 24%); moreover, the costs of revision prostheses were almost threefold higher than those of primary prostheses. Therefore, whether the financial status of patients is considered when selecting primary or revision prostheses should be investigated. In Taiwan, medical expenditure is guided by National Health Insurance (NHI), and the cost of each component of revision TKA is based on NHI points in National Health Insurance Fee Schedule (Table 5). Because of the cost of stem rods and wedge augments, primary prostheses with tibial stems would be more expensive than primary prostheses, and the cost of revision prostheses would be the highest. Therefore, considering the medical cost, a primary prosthesis or primary prosthesis with a tibial stem remains the first choice if the condition is suitable.

**Table 5:** Cost of each component of revision TKA in Taiwan

Revision component	Zimmer^1^		United^1^	
	Cost^2^	NHI points^3^	Cost^2^	NHI points^3^
TKA	80450	64704	69000	64704
Femoral component	38750	19106.85	20336	19106.85
Tibial component	23000	14732.55	16000	14732.55
Insert	26837	26265.75	26837	26265.75
Patella	7100	6259.05	6662	6259.05
Stem rod	8700	5966.1	7000	5966.1
Femoral augment	4400	3138.45	3900	3138.45
Tibial wedge	4400	3138.45	3900	3138.45

^1^ The most common brands of revision TKA prostheses in Taiwan are Zimmer and United

^2^ The cost is presented as New Taiwan Dollar

^3^ NHI points are based on National Health Insurance Fee Schedule in Taiwan

### Strengths and limitations

This study has several limitations. First, this was a retrospective study with a small sample size and high percentage of excluded patients due to loss of data or follow up. Second, the conversion of a failed UKA can be a technically demanding procedure that depends on how conservative the initial procedure was and the failure mode [16]. Although we developed surgical steps for converting UKA to TKA, we lacked information on each surgeon’s technique; moreover, information on the indication for the previous UKA performed in other hospitals was unavailable. Therefore, the interpretation of survival outcomes is challenging. Technique-dependent factors cannot be neglected for surgeries performed by different surgeons.

## Conclusions

The most common cause for UKA failure was aseptic loosening in this study. The adoption of a standardized surgical technique makes it easier to perform revision surgeries. Compared with primary prostheses, primary prostheses with tibial stems provided higher stability, leading to a lower failure rate due to less risk of aseptic loosening in patients with tibial AORI grade 2a. In patients with tibial AORI grade 1, bone defects were absent; therefore, aseptic loosening and failure of revision surgery were not noted in any groups. Moreover, primary prostheses with tibial stems were more affordable than revision prostheses and involved a smaller degree of surgical invasion, thereby lowering the risk of infection. Although small sample size and significant loss of data are the limitation of this study, we report the surgical outcomes in our experience and we think it is still though valuable. We advise surgeons may try using primary prostheses in patients with tibial AORI grade 1 and primary prostheses with tibial stems in patients with tibial AORI grade 2a.

## Data Availability

Not applicable.

## Data Availability

All data generated during this study are included in this article.
